# Preliminary Evidence of the Possible Roles of the Ferritinophagy-Iron Uptake Axis in Canine Testicular Cancer

**DOI:** 10.3390/ani14172619

**Published:** 2024-09-09

**Authors:** Rebecca Leandri, Karen Power, Sara Buonocore, Gionata De Vico

**Affiliations:** Department of Biology, University of Naples ‘Federico II’, Via Vicinale Cupa Cinthia 21, 80216 Napoli, Italy; rebecca.leandri@unina.it (R.L.); sara.bcore@gmail.com (S.B.); gionata.devico@unina.it (G.D.V.)

**Keywords:** iron metabolism, ferritinophagy, FTH1, NCOA4, seminoma, Sertoli cells, spermatogenesis, TfR1

## Abstract

**Simple Summary:**

This study presents data on the immunohistochemical expression of key iron metabolism proteins in non-neoplastic and neoplastic canine testes. Iron is crucial for spermatogenesis, and its regulation in testicular cells is essential for normal function. This study confirms that Sertoli cells and Transferrin Receptor 1 (TfR1) play significant roles in iron uptake in dogs, aligning with findings in humans and mice. However, it highlights a unique expression of nuclear receptor coactivator 4 (NCOA4) in canine Sertoli cells, suggesting a specific role in iron recycling through ferritinophagy. Differences in iron metabolism were observed in various canine testicular tumors. Higher TfR1 and NCOA4 expressions were noted in Leydig cell tumors and diffuse seminomas, indicating a reliance on iron for tumor growth. The altered expression of iron-related proteins in tumors underscores their potential as therapeutic targets. Targeting TfR1 and utilizing iron-modulating therapies shows promise. Further research is needed to explore the therapeutic potential of modulating iron metabolism in canine testicular cancers.

**Abstract:**

Iron is a key element in spermatogenesis; its metabolic pathway in the testis is strictly regulated. Alterations in iron metabolism are linked to various diseases, including cancer, and changes in iron metabolism-related proteins have been observed in multiple human, mouse and canine tumors. There is limited knowledge about iron metabolism in canine non-neoplastic and neoplastic testes. This study aimed to explore the immunohistochemical expression of molecules involved in iron uptake and storage [Transferrin Receptor 1 (TfR1), ferritin (FTH1), nuclear receptor coactivator 4 (NCOA4)] and PCNA in canine non-neoplastic and neoplastic testicular samples. Non-neoplastic testes showed moderate TfR1 expression in developing germ cells and Sertoli cells, high NCOA4 cytoplasmic immunostaining in the Sertoli cells and occasional cytoplasmic immunopositivity for FTH1 in the spermatogonia and Sertoli cells. In contrast, Leydig cell tumors (LCTs) and Diffuse Type Seminoma (DSEM) exhibited increased expression of TfR1, along with higher PCNA expression, suggesting a higher iron need for proliferation. Intratubular Type Seminoma (ITSEM) showed a higher FTH1 expression, indicating greater iron storage, while the increased NCOA4 expression in the LCTs and DSEM suggested ferritinophagy to release iron for proliferation. Sertoli cell tumors (SCTs) showed only NCOA4 expression. These preliminary findings highlight potential molecular targets for developing new anti-neoplastic treatments in canine testicular tumors.

## 1. Introduction

Iron is an essential constituent and cofactor of several enzymes and proteins involved in cellular processes such as electron transport, respiration, DNA synthesis and cell division [1]. Spermatogenesis is an iron-dependent process [2], and iron metabolism is strictly regulated in the testis [3]. Sertoli cells (SCs) drive iron from the basal to the ad luminal compartment of the testis through transferrin (Tf), a plasma glycoprotein that binds two specific sites of ferric iron (Fe^3+^). Tf is internalized by Transferrin Receptor 1 (TfR1), a membrane glycoprotein located on the basolateral SC membrane [4]. The Tf-TfR1 complex also occurs in spermatogonia and primary spermatocytes where iron is internalized to support mitotic divisions, DNA synthesis and mitochondriogenesis. If not immediately used, iron is stored in ferritin (Ft) cages [5], consisting of a combination of heavy (H)-chain and light (L)-chain ferritin types. Ft degradation and consequent iron release can occur by ferritinophagy, a process mediated by nuclear receptor coactivator 4 (NCOA4) [6], which binds ferritin heavy chain 1 (FTH1), starting the autophagic degradation of the Ft shell [7].

Iron is then excreted from the spermatozoa as part of the residual bodies, which are phagocyted by SCs and stored again in the Ft. Finally, iron release occurs from the SCs, where it can be exported in the bloodstream by ferroportin (FPN) [3,8]. Also, a little portion of iron carried by the spermatozoa leaving the seminiferous tubules is soon replaced by the Tf-TfR1 complex located on the SC basolateral membrane, ready to start the cycle again. In physiological conditions, iron uptake and ferritinophagy are regulated by intracellular iron levels, which either encourage or inhibit the processes based on the availability and requirement of iron [9]. Iron metabolism is strictly regulated both at the systemic and cellular levels, and it involves a set of interconnected regulatory pathways involving the hepcidin–ferroportin axis, hypoxia-inducible factor 2α (HIF2α) and Iron Regulatory Proteins (IRPs), which control the absorption, mobilization and excretion of iron following its fluctuations in blood and tissues and increased/decreased cell requirements [10,11,12].

However, dysregulation of the iron metabolism has been observed in various diseases such as cancer [13,14,15,16,17]. The accumulation of intracytoplasmic free iron can lead to the production of Reactive Oxygen Species (ROS) through Fenton reactions [18]. Excessive ROS can in turn cause oxidative stress, impair metabolic functions and damage essential cell macromolecules such as proteins, lipids and DNA, inducing genetic instability, which can be found at the onset of tumors [19,20]. Moreover, increasing iron uptake and reducing its release from tumor cells appears to support their survival; rapid-division cancer cells present a higher need for iron compared with normal cells to sustain higher rates of proliferation [13,21,22] and invasion of surrounding tissues [23]. For these reasons, compared with non-cancerous cells, cancer cells are more reliant on iron and are more vulnerable to iron depletion, a condition referred to as “iron addiction” [1]. The greater requirement of iron by neoplastic cells seems to be satisfied by altered expression of iron metabolism-related proteins such as TfR1, Ft and NCOA4, as detected in human pancreatic, liver, breast, prostate, leukemia, ovarian, lung and colon tumors [14,16,22,24,25]. Functional studies involving knockdown, knockout or overexpression of TfR1, Ft and NCOA4 have further confirmed the importance of these proteins in cancer progression. Regarding TfR1, a study by Feng et al. (2023) has shown the effects of TFRC knockdown in the progression of nasopharyngeal carcinoma (NPC). Indeed, TFRC knockdown decreased intracellular iron and intracellular FTH1 and inhibited cell proliferation in NPC cell lines [26]. Moreover, downregulation of TfR1 expression inhibited cell proliferation, promoted cells from the G1 phase to the S phase and facilitated cell migration and invasion in colonorectal cancer [27]. In a study by Bellelli et al. (2016), NCOA4-null mice were shown to develop tissue accumulation of Ft and iron; moreover, in primary embryonic fibroblasts obtained from NCOA4-null mice, impaired ferritinophagy and ferritin accumulation were observed consequently to impaired autophagic targeting [28]. Also, a study on human pancreatic cancer showed that NCOA4 knockout xenografts presented marked tumor growth delay, and PDAC cell lines with NCOA4 depletion after knockdown or clonal knockout demonstrated a marked growth delay due to a significant decrease in free iron [16]. Moreover, NCOA4 knockout HeLA cells presented abolished ferritinophagy and increasing ferritin levels, while overexpression of NCOA4 caused an induction of Ft lower than that of the control HeLa cells [29].

In the canine species, studies on the expression of iron metabolism-related proteins are restricted to lymphomas, malignant oronasal tumors, brain tumors, osteosarcomas and hepatocellular carcinoma [30,31,32,33,34,35]. To date, no study has focused on the expression of iron-related proteins in testicular tumors and their normal counterparts, although alterations of iron metabolism have been previously described in humans [36,37,38,39].

The canine testis is the fourth most frequent site of tumor development [40], and different histotypes can be recognized: Sertoli cell tumors (SCTs) and Leydig cell tumors (LCTs) derive from sex cord stromal cells [41,42] while seminomas (SEMs) develop from germ cells. According to the WHO histological classification of tumors of domestic animals [41], seminomas can be classified as intratubular (ITSEMs) or diffuse (DSEMs). Canine testicular tumors show high proliferation rates [43]. However, some differences have been reported: SCTs and LCTs show lower proliferation rates compared to SEM [44]. The aim of our study was to preliminarily investigate the expressions of TfR1, NCOA4, FTH1 and PCNA in canine non-neoplastic and neoplastic testicles to understand if the proliferative process could be connected to higher iron requirements and altered iron metabolism.

## 2. Materials and Methods

### 2.1. Animals

Twenty-six testicular specimens coming from 26 different individuals were collected from the archives of the Department of Biology—University of Naples Federico II. All sampling procedures were performed for diagnostic purposes; thus, this study did not require ethical approval according to European Directive 2010/63/EU. Age and breed information for all dogs were available and are reported in Table S1 of the Supplementary Materials.

### 2.2. Histology

Specimens were fixed in 10% neutral buffered formalin and then processed for routine histolopatology; 3 μm sections were cut from paraffin blocks, stained with hematoxylin and eosin (H&E) and observed by light microscopy. Histotype diagnosis was performed by an experienced pathologist (GDV) according to the guidelines proposed by the World Health Organization [41].

### 2.3. Immunohistochemistry

Additional 3 μm sections were mounted on positively charged glass slides (Bio-Optica, Milan, Italy), deparaffined in xylene, rehydrated in decreasing series of alcohol, and endogenous peroxidase activity was blocked with a solution of hydrogen peroxide and methanol (4:1) for 20 min. Following a rinse in distilled water, heat-induced antigen retrieval was performed with a citrate buffer (pH 6) in a microwave oven (650 W, high power) for 6 min. Immunohistochemistry was performed according to the protocol suggested by the MACH1 Universal HRP-Polymer Detection Kit (Cat. No: M1U539 G, L10; Bio-Optica, Milan, Italy). The sections were blocked with a protein block (MACH1; Biocare Medical LLC., Concord, CA, USA) for 60 min. Then, the slides were incubated overnight at 4 °C with the primary antibody diluted in phosphate-buffered saline (PBS) (0.01 M PBS, pH 7.4). The dilutions are reported in Table 1. In the corresponding negative control sections, the primary antibody was omitted. To reveal immunolabeling, diaminobenzidine tetrahydrochloride was used as a chromogen and sections were counterstained in Mayer’s hematoxylin. To evaluate the specificity of the markers, replicate sections were incubated with isotype-specific immunoglobulins and used as negative controls [45]. Slides were examined and photographed with a light microscope (AXIO SCOPE.A1, Carl Zeiss S.p.A., Oberkochen, Germany) equipped with a microphotography-system digital camera (Axiocam 105 color, Carl Zeiss S.p.A., Oberkochen, Germany). Immunolabeling for each antibody in each sample was scored by two independent observers (RL and KP) under blinded conditions. The number of positively labeled cells was determined by counting 1.000 cells in 10 fields at 400× magnification (40× objective 10× ocular) and expressed as a percentage.

Immunoreactivity was scored from none to high, as in previous studies [46,47,48,49]. No cells labeled was indicated as “-” and scored as 0; occasional labeling (<10%) was indicated as “+” and scored as 1; a low percentage of labeled cells (11–40%) was indicated as “++” and scored as 2; a moderate percentage of labeled cells (41–80%) was indicated as “+++” and scored as 3; and a high percentage (81–100%) of labeled cells was indicated as “++++” and scored as 4.

### 2.4. Western Blot

To test the specificity of the IHC signals, a Western blot was performed. A small portion of the tissues from the testicular specimens was collected to extract the total protein. The samples were lysed by using an ice-cold RIPA buffer (150 mM sodium chloride, 1% Triton X-100, 0.5% sodium deoxycholate, 0.1% sodium dodecyl sulfate, 50 mM Tris, pH 8.0) supplemented with an inhibitor cocktail (78420, ThermoFischer Scientific, Carlsbad, CA, USA) and 1 mM PMSF. The total concentration of protein was determined by using a NanoDrop 1000 spectrophotometer (ThermoFisher Scientific, Carlsbad, CA, USA). In total, 60 µg of proteins of testicular specimens were separated by sodium dodecyl sulfate–polyacrylamide gel electrophoresis (SDS–PAGE) and transferred to PVDF membranes (IPVH00010, EMD Millipore, Darmstadt, Germany,), then blocked in Tween 0.1% Tris-buffered saline containing 5% non-fat milk (70166, Merck Millipore, Darmstadt, Germany,) for 1 h at room temperature and subsequently incubated overnight at 4 °C with primary antibodies. The antibodies and their relative dilutions used for the Western blot analysis are reported in Table 2. After washing with 0.1% TBS, the membranes were incubated for 1 h at room temperature with the following secondary antibodies: goat anti-rabbit IgG antibody, HPR conjugate and goat anti-mouse IgG antibody and HPR conjugate (1:5000 EMD Millipore, 12–348; 12–349). Following an incubation with an Enhanced Chemiluminescence solution (Clarity Western ECL substrate, 1705061, Bio-Rad, Hercules, CA, USA) for 5 min at RT, the horseradish peroxidase-conjugated secondary antibody generated a chemiluminescent signal detected by the XRS+ Chemidoc Imaging System (Bio-Rad, Hercules, CA, USA). Biological duplicates were performed to ensure the reproducibility of the results.

### 2.5. Statistical Analysis

In this study, a statistical analysis was conducted using a one-way analysis of variance (ANOVA) to determine if there were any significant differences among the expressions of the iron-related proteins under investigation between the various tumor type samples (SCTs, ITSEMs, DSEMs and LCTs) and non-neoplastic samples. A *p*-value of less than 0.05 was considered statistically significant.

## 3. Results

### 3.1. Histological Results

Non-neoplastic testes were detected in 11.5% of the analyzed testes and used as controls, while testicular tumors were detected in 88.4%. Specifically, ITSEM was diagnosed in 19.2% of dogs (21.7% of tumors), DSEM in 42.4% of dogs (47% of tumors), SCTs in 19.2% of dogs (21.7% of tumors) and LCTs in 7.7% (8.6 of tumors). A comprehensive distribution of the histological findings is reported in Table 3.

**Figure 1:**
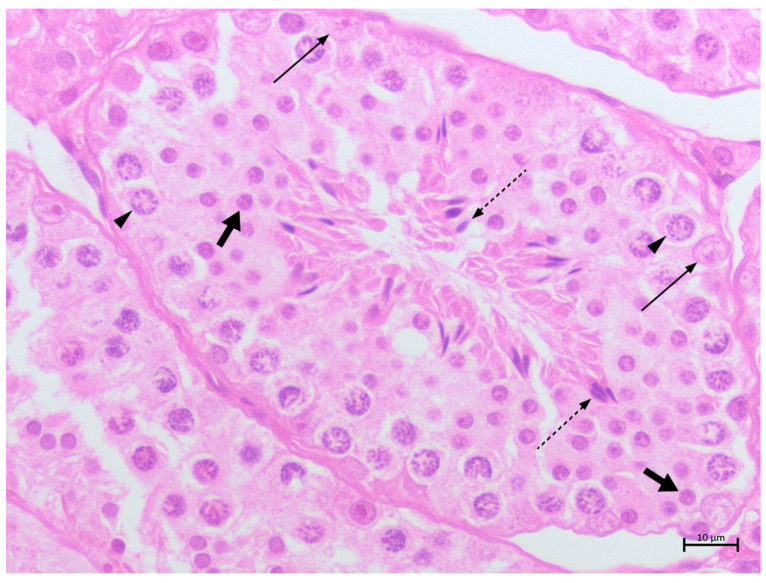
Canine testis. Non-neoplastic showing complete spermatogenesis. Sertoli cells (thin arrows), spermatocytes I/II (arrow heads), spermatids (fat arrows) and spermatozoa (dotted arrow) (H&E stain 40×, BAR 10 μm).

In the non-neoplastic samples, spermatogenesis appeared complete and fully formed seminiferous tubules contained SCs with irregular highly folded chromatin and prominent nucleoli. In the interstitium, the LCs were generally barely visible and characterized by abundant pink vacuolized cytoplasm and round nuclei with distinct nucleoli (Figure 1).

**Figure 2:**
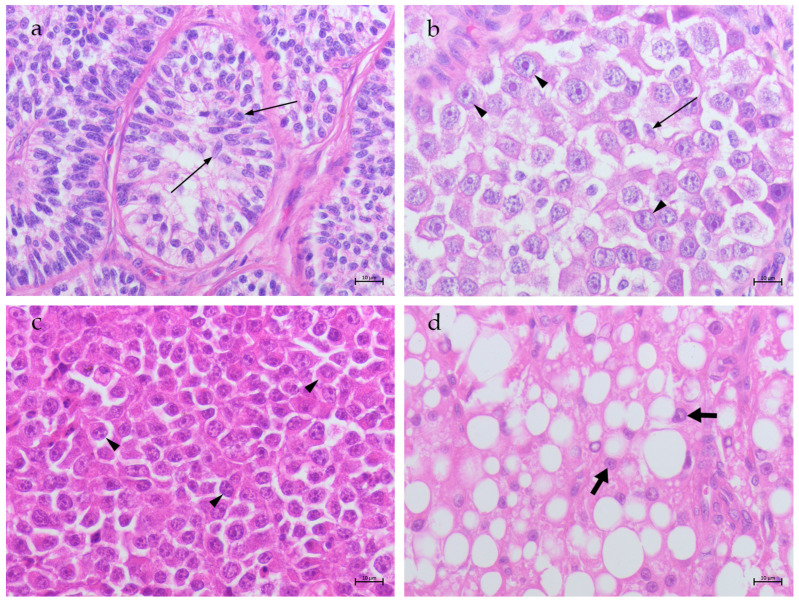
Canine neoplastic testes. (**a**) SCT: presenting tubules lined by neoplastic Sertoli cells (thin arrows); (**b**) ITSEM: seminiferous tubules containing big neoplastic cells (arrow heads) and a few Sertoli-like cells (thin arrow); (**c**) DSEM: sheets of small round/oval neoplastic cells (arrow heads); and (**d**) LCT: showing polygonal vacuolized cells (fat arrows) arranged in cords separated by stroma (H&E stain. 40×, BAR 10 μm).

The SCTs were characterized by spindle-shaped cells with oval hyperchromatic nuclei that contained prominent nucleoli. The cytoplasms varied from eosinophilic to pale (Figure 2a). The ITSEMs were characterized histologically by cells with big, round nuclei, finely granular chromatin and multiple prominent nucleoli. Moderate in quantity and pale to clear, the cytoplasm was present. Intermingled residual Sertoli-like cells, characterized by small nuclei, prominent nucleoli and flame-shaped cytoplasm, were detected within the seminiferous tubules (Figure 2b). The DSEMs were characterized by a number of small oval/round cells with abundant cytoplasm, prominent nuclei and single or multiple nucleoli (Figure 2c). The LCTs showed polygonal cells with abundant eosinophilic, highly vacuolized cytoplasm; uniform round nuclei; and prominent central nucleoli (Figure 2d).

### 3.2. Immunohistochemical Results

For detailed immunoscoring of each antibody, see Table 3.

#### 3.2.1. Non-Neoplastic Testes

**Figure 3:**
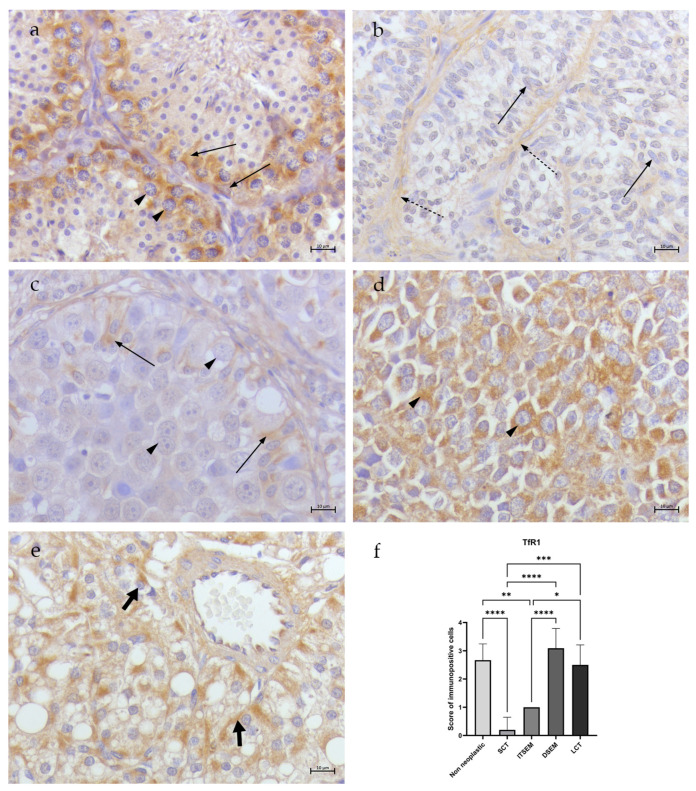
TfR1 immunohistochemistry of canine non-neoplastic and neoplastic testes. (**a**) Non-neoplastic testis. Moderate cytoplasmatic TfR1 immunostaining in all spermatogonia, Sertoli cells (thin arrows) and spermatocytes I (arrow heads). (**b**) SCT. No signal for TfR1 immunostaining in Sertoli cells (thin arrows) within the tubules; occasional immunopositivity only in interstitial cells (dotted arrows). (**c**) ITSEM. No signal in tumoral cells (arrow heads) and occasional cytoplasmatic TfR1 immunostaining in residual Sertoli cells (thin arrow). (**d**) DSEM. Moderate/high cytoplasmatic TfR1 immunostaining in neoplastic cells (arrow heads). (**e**) LCT. Moderate cytoplasmatic TfR1 immunostaining in Leydig cells (fat arrows). (**f**) Statistical analysis of TfR1 expression—*: *p* < 0.05, **: *p* < 0.01, ***: *p* < 0.001, ****: *p* < 0.0001 (40×, BAR 10 μm).

**Figure 4:**
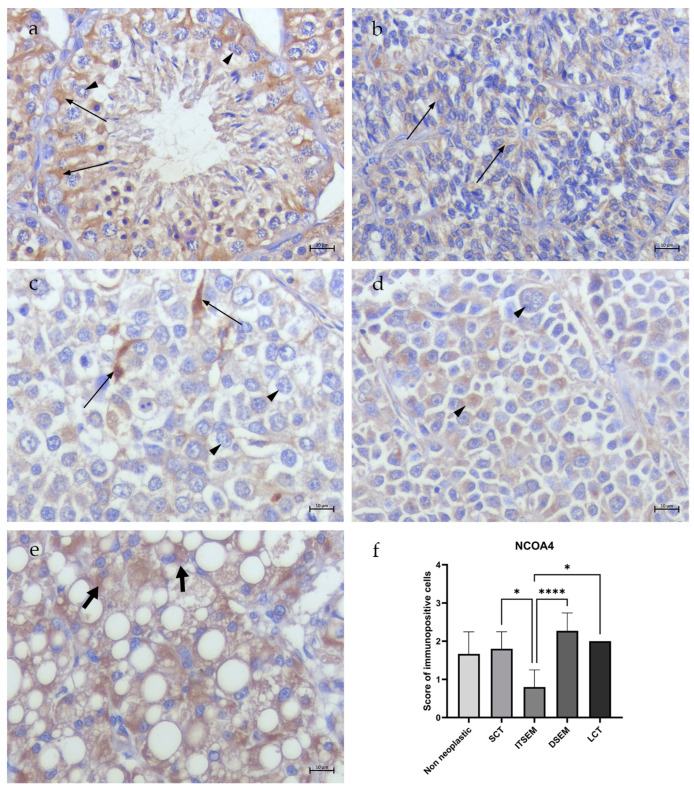
NCOA4 immunohistochemistry of canine non-neoplastic and neoplastic testes. (**a**) Non-neoplastic testis. High NCOA4 cytoplasmatic immunostaining in Sertoli cells (thin arrows) and no signal in germinal cells (arrow heads). (**b**) SCT. Low signal for NCOA4 immunopositivity in Sertoli cells within the tubules (thin arrows). (**c**) ITSEM. No immunostaining in tumoral cells (arrow heads) and occasional cytoplasmatic NCOA4 immunostaining revealed in residual Sertoli cells (thin arrows). (**d**) DSEM. Low cytoplasmatic NCOA4 immunostaining in neoplastic cells (arrow heads). (**e**) LCT. Moderate cytoplasmatic immunostaining for NCOA4 in Leydig cells (fat arrows); (**f**) Statistical analysis of NCOA4 expression—*: *p* < 0.05, ****: *p* < 0.0001 (40×, BAR 10 μm).

**Figure 5:**
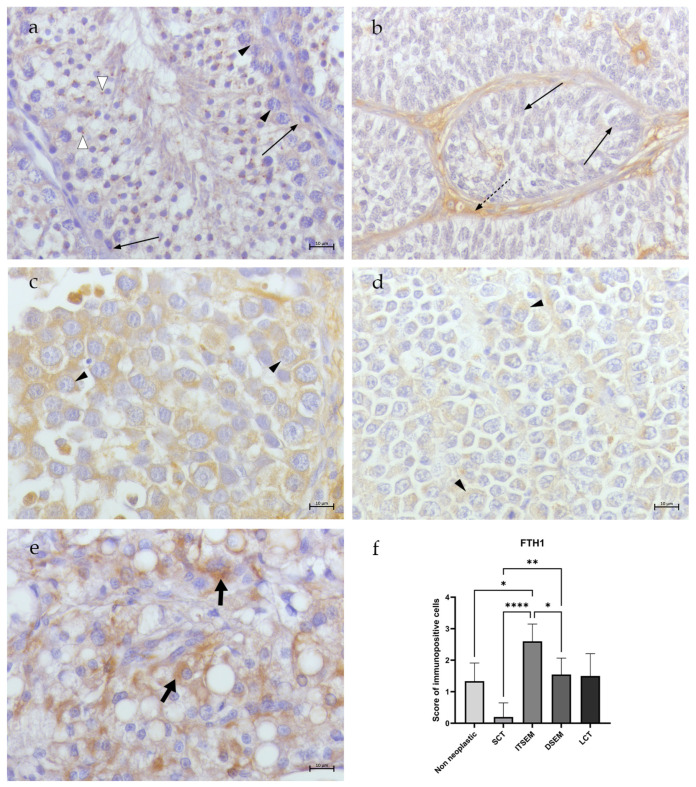
FTH1 immunohistochemistry of canine non-neoplastic and neoplastic testes. (**a**) Non-neoplastic testis. Occasional cytoplasmatic immunopositivity for FTH1 in spermatogonia and spermatocytes I (arrow heads) and Sertoli cells (thin arrows); some labeled perinuclear dots in spermatocytes II (white arrow heads). (**b**) SCT. No signal for FTH1 immunostaining in Sertoli cells (thin arrows) within the tubules; occasional immunopositivity in interstitial cells (dotted arrow). (**c**) ITSEM. Moderate cytoplasmatic immunopositivity for FTH1 in neoplastic cells (arrow heads). (**d**) DSEM. Occasional/low cytoplasmatic immunopositivity for FTH1 in neoplastic cells (arrow heads). (**e**) LCT. Occasional/low cytoplasmatic immunopositivity for FTH1 in Leydig cells (fat arrows). (**f**) Statistical analysis of FTH1 expression—*: *p* < 0.05, **: *p* < 0.01, ****: *p* < 0.0001 (40×, BAR 10 μm).

**Figure 6:**
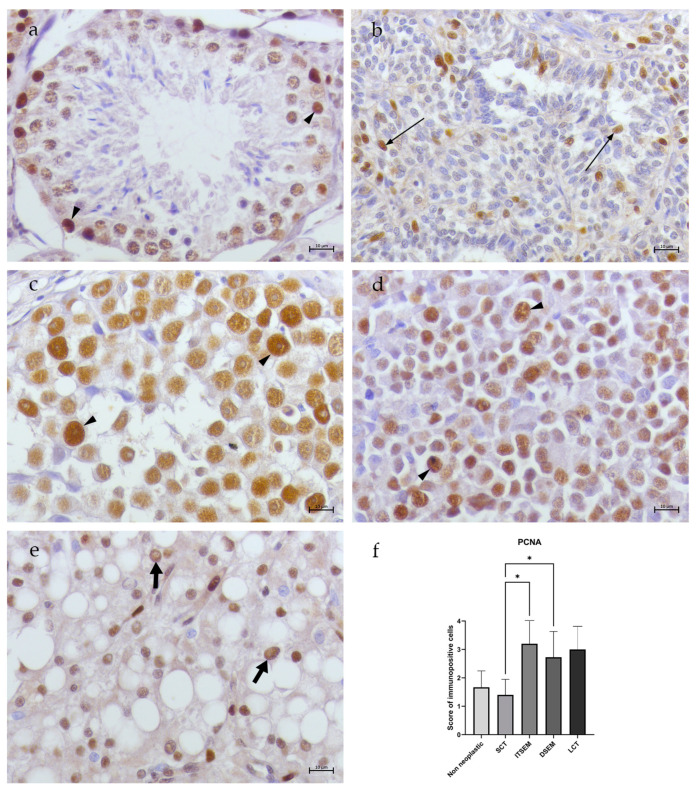
PCNA immunohistochemistry of canine non-neoplastic and neoplastic testes. (**a**) Non-neoplastic testis. Moderate immunostaining for PCNA in spermatogonia and spermatocyte I nuclei (arrow head). (**b**) SCT. Occasional immunostaining for PCNA in Sertoli cell nuclei (thin arrow). (**c**) ITSEM. Moderate immunostaining for PCNA in neoplastic cell nuclei (arrow heads). (**d**) DSEM. Moderate immunostaining for PCNA in neoplastic cell nuclei (arrow heads). (**e**) LCT. Moderate immunostaining for PCNA in Leydig cell nuclei (fat arrows). (**f**) Statistical analysis of PCNA expression—*: *p* < 0.05, (40×, BAR 10 μm).

In all non-neoplastic samples (N1–N3), the totality of spermatogonia, spermatocytes I and Sertoli cells showed low/moderate (scoring of 2.6 ± 0.57) cytoplasmic TfR1 immunostaining (Figure 3a). Occasional/low NCOA4 immunostaining (scoring of 1.6 ± 0.57) was detected only in the Sertoli cell cytoplasm, while no signal was revealed in the germ cells (Figure 4a). Occasional cytoplasmatic FTH1 immunopositivity (scoring of 1.3 ± 0.57) was detected in the spermatogonia and Sertoli cell membranes, while several spermatocytes I/spermatocytes II presented perinuclear labeling (Figure 5a). Moreover, occasional/low PCNA immunopositivity (scoring of 1.6 ± 0.57) at the nuclear level was detected only in the spermatogonia and spermatocytes I (Figure 6a).

#### 3.2.2. Sertoli Cell Tumors

In the SCT samples (S1–S5), NCOA4 immunopositivity was detected in the Sertoli cells in an occasional/low percentage (scoring of 1.8 ± 0.44) (Figure 4b), while no signal of TfR1 (Figure 3b) and FTH1 positivity was detected in the Sertoli cells within the tubules (scoring of 0.2 ± 0.44) (Figure 5b). An occasional signal was observed only in some interstitial cells. Moreover, occasional/low signals for PCNA (scoring of 1.4 ± 0.54) positivity in the Sertoli cell nuclei were revealed (Figure 6b).

#### 3.2.3. Seminomas

In the ITSEM samples (S6–S10), very occasional TfR1 (scoring of 1) (Figure 3c) and NCOA4 immunostaining (scoring of 0.8 ± 0.44) was spotted only in the Sertoli cell cytoplasm (Figure 4c) while low/moderate cytoplasmatic immunostaining for FTH1 (scoring of 2.6 ± 0.54) was revealed (Figure 5c). In the ITSEMs, the majority of neoplastic cells within the tubules were intensively labeled at the nuclear level by PCNA (scoring of 3.2 ± 0.83) (Figure 6c). On the contrary, in the DSEM samples (S11-S21), moderate/high TfR1 immunostaining (scoring of 3 ± 0.7) was revealed in the cytoplasm of almost the totality of the neoplastic cells (Figure 3d), whereas low NCOA4 immunostaining (scoring of 2.27 ± 0.46) (Figure 4d) and occasional/low immunopositivity for FTH1 (scoring of 1.54 ± 0.52) were detected in the neoplastic cell cytoplasm (Figure 5d). The PCNA was positive in the majority of neoplastic cell nuclei (scoring of 2.63 ± 1.02) (Figure 6d).

#### 3.2.4. Leydig Cell Tumors

In the LCT samples (S22–S23), the Leydig cells showed low/moderate cytoplasmatic immunostaining for TfR1 (scoring of 2.5 ± 0.70) (Figure 3e) and NCOA4 (scoring of 2) (Figure 4e), whereas occasional/low cytoplasmatic staining for FTH1 (scoring of 1.5 ± 0.7) was detected (Figure 5e). An increased PCNA immunoreactivity compared to the non-neoplastic testes was also reported in the LCT samples, where Leydig cells were labeled by PCNA in the nuclei in a moderate way (scoring of 3) (Figure 6e).

### 3.3. Western Blot Results

**Figure 7:**
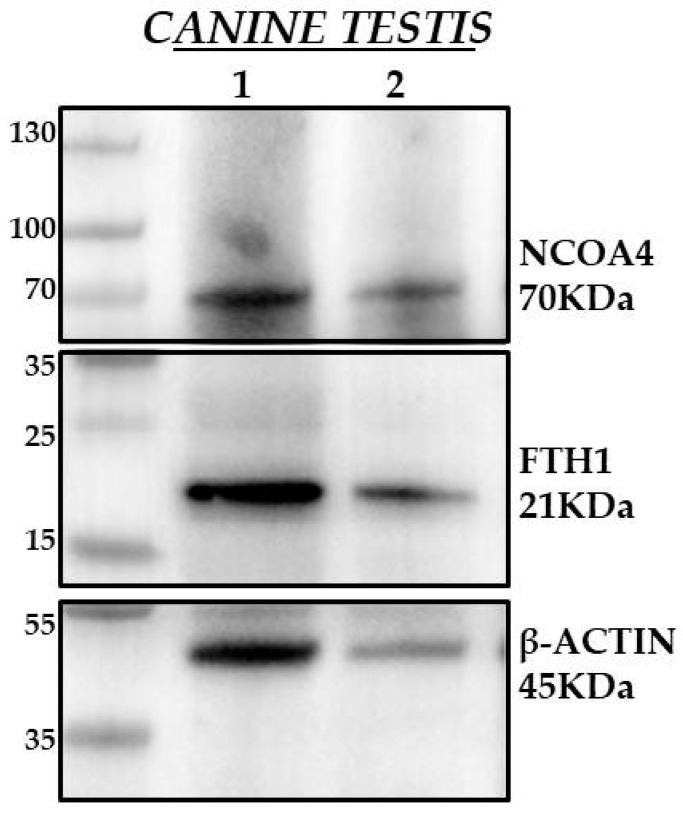
Representative NCOA4 and FTH1 immunoblot analysis in non neoplastic canine testis. Immunoblot analysis performed on the total protein lysates of two non-neoplastic canine testis samples. In total, 60 µg of lysates were loaded for each sample, the tag of which is reported on the right of the membrane. The membranes were cut using the protein ladder as a reference and incubated with the primary anti-NCOA4 antibody and anti-FTH1 antibody. The immunoreactive band against β-Actin was used as a loading control.

Western blot analysis was performed to check the specificities of the mouse anti-NCOA4 and rabbit anti-FTH1 antibodies on the total protein lysates from the non-neoplastic canine testis. As displayed in Figure 7, the NCOA4-specific antibody gave an immunoreactive band of ~70 KDa in both total protein lysates of the canine testis, showing that this antibody recognizes the canine isoform of NCOA4 at the right molecular weight, as expected, since the NCOA4 gene codes a 614-amino acid protein with an estimated molecular weight of 70 KDa [50]. Similarly, the incubation of the membranes carrying protein lysates of canine testes with the primary antibody FTH1 displayed a ~20 KDa protein band (Figure 7), confirming that this primary antibody specifically targets the 21 KDa FTH1 protein. Altogether, these results demonstrate for the first time the specificity of these two primary antibodies into selective immunoreactions with FTH1 and NCOA4 in dogs. Additional information regarding protein loading and the significance of the results can be found in Figure S1.

### 3.4. Statistical Analysis Results

The results showed various significant differences between the study groups. Regarding TfR1 (Figure 3f), there was a noteworthy statistically significant difference (****) between the SCTs and ITSEMs compared to the non-neoplastic group, as both tumor types exhibited lower expression of TfR1. In contrast, the DSEMs and LCTs showed higher expression levels of TfR1, although these were not statistically significant when compared to the non-neoplastic group. In a tumor context, the most significant differences (****) were seen between the ITSEMs and DSEMs, highlighting the difference in iron uptake between the two types of seminomas. The SCTs showed statistically significant differences compared to every group analyzed except for the ITSEMs. As concerns NCOA4 expression, (Figure 4f), there were no statistically significant differences compared to the control; meanwhile, within the tumor context, the ITSEMs showed significantly lower expression compared to all other tumor types, particularly to the DSEMs (****). Additional results highlighting iron-related protein expression alterations in the ITSEM group were shown by FTH1 expression (Figure 5f). Indeed, the ITSEM group showed the most significant differences compared with the other groups, with a very high significance when compared to the SCTs (****) and non-neoplastics (*). There were also significant differences between the ITSEMs and LCTs (*) and between the ITSEMs and DSEMs (*). In the analysis of the proliferation marker PCNA (Figure 6f), statistically significant differences were observed between the ITSEMs and all other groups except for the LCTs. Notably, there was a highly significant difference between the ITSEMs and SCTs (****). Additionally, a significant difference was also found between the DSEMs and SCTs (**), further highlighting the lower expression of PCNA in SCTs compared with other tumor types. These findings underscore the distinct proliferative profiles of ITSEM and DSEM compared to SCTs.

## 4. Discussion

In the past years, iron and its metabolism have been gaining more and more importance in the research field due to their role in cancer development and progression [51]. In this preliminary study, we analyzed, for the first time, the immunohistochemical expression of key molecules involved in iron metabolism in non-neoplastic and neoplastic canine testes. In humans [5], a central role of iron recycling within and between seminiferous tubule cells (SCs/germ cells) has emerged as a regulatory element of normal spermatogenesis [52]. Our results propose the idea that in dogs as well, iron circulation in the physiological testis could be constitutively involved in normal spermatogenesis, suggesting a central role of SCs, TfR1 and ferritinophagy in the process. In line with previous results in both humans and mice [50,53,54], our study showed TfR1 immunolabeling in spermatogonia and primary spermatocytes, supporting the key role of TfR1 in iron uptake and internalization in non-neoplastic developing canine germ cells. We also observed TfR1 expression in non-neoplastic SCs. Previous studies have reported contrasting results, confirming [3,5,53,54] or contradicting our findings [3,55]; therefore, there is a need for further studies aimed at evaluating the role of TfR1 in iron loading into canine SCs in a wider number of samples to be performed. Moreover, NCOA4 expression in non-neoplastic canine testis appeared to be high but limited to SCs or “nurse cells”, suggesting a significant release of bioavailable iron ready to be transferred to germ cells to support the replication processes [56]. Also, rapid turnover of iron in seminiferous tubule cells is supported by the weak Ft immunolabeling detected in the SCs, suggesting their reduced need for iron storage. This finding could be connected to the crucial role of SCs in phagocyting iron from spermatozoa and rapidly restoring the iron cycle [3], probably to support the rapid proliferation suggested by PCNA immunopositivity [57]. Interestingly, FTH1 positivity was also detected in spermatids as perinuclear dots, suggesting an increased need for iron in the mitochondria, which are located in a peculiar cellular position [58], to sustain the increased metabolic rate during spermatogenesis [59].

In the testicular tumors, the expressions of TfR1, FTH1 and NCOA4 were altered compared to the non-neoplastic samples. The TfR1 expression was markedly higher in the LCTs and diffuse SEMs; meanwhile, in the ITSEMs, the germ cells were negative while some cells resembling SCs were found positive. This result is interesting in the context of the reciprocal inductive relationship existing between SCs and germ cells during SEM development. A previous study of humans by Fink et al. [60] showed that in precursor lesions of testicular germ-cell tumors such as germ-cell neoplasia in situ (GCNIS), SCs acquire a state of undifferentiation postpubertally and consequently fail to support the process of spermatogenesis [61]. SCs’ progressive dedifferentiation in this lesion could promote the proliferation and progression of neoplastic cells from puberty onward [60]. Our results suggest a similar process in canine Sertoli-like cells in ITSEM, showing very low TfR1 expression and occasional NCOA4 expression compared to non-neoplastic testes, hinting at a loss of Sertoli-like cell function. Further studies to confirm the dedifferentiation of these cells in canine ITSEM are needed. Nevertheless, the Sertoli-like cells showed a higher FTH1 expression, the function of which remains unclear. Interestingly, decreased TfR1 expression and increased FTH1 expression were observed in the ITSEM germ cells, suggesting the presence of a second iron uptake pathway in this type of tumor, which should be further investigated in future studies.

In the DSEMs and LCTs, NCOA4 expression was considerably higher than in other canine testicular tumor types, while the expression of FTH1 appeared lower. Our results confirmed the inverse association between the presences of NCOA4 and FTH1, suggesting also that in some canine testicular tumors, the activation of the “ferritinophagic” process to release higher amounts of iron is needed to sustain the higher proliferative rate of neoplastic cells [62,63], as confirmed by higher PCNA expression in these types of tumors.

In the SCTs, the iron metabolism appeared completely altered. The tumoral SCs did not reveal positivity for TfR1 nor FTH1, suggesting loss of their function as “nurse cells”. Indeed, iron proteins are generally used to sustain germinal cell proliferation and not SC replication, since they are nondividing cells [64] in physiological conditions. However, as SCTs are characterized by low proliferation rates [43], confirmed by PCNA expression only in a few neoplastic SCs, our results showed that NCOA4-driven ferritinophagy is enough to support proliferation in this type of tumor. Altogether, our results suggest the occurrence of alterations of the iron metabolism in the tumoral seminiferous tubules; however, the limited number of samples and the variety of the histotypes analyzed represent two issues that could compromise the generalization of the results. They should instead be considered as a starting point for future research comprising a wider sample number and greater representability of each histotype group.

Alteration of the iron metabolism has been already described in different diseases concerning the human male reproductive system [5,37,65]. Considering that the canine species can be regarded as an effective spontaneous model for human cancer, and, even more importantly, that dogs and humans share the same histotypes of testicular tumors according to the WHO [41], we believe that our results could also trigger future studies on the expression of iron-related proteins in the human tumoral seminiferous tubule.

The altered expression of iron-related proteins in almost all of the canine testicular neoplasia highlights their significance as potential therapeutic targets [14,33]. Studies have shown that targeting TfR1 is crucial for antibody-mediated therapy, as the use of cytotoxic antibodies can directly disrupt TfR1 function, inhibit cell growth and induce apoptosis [66]. TfR1 could also be used to increase the selectivity of treated cells by delivering therapeutic molecules through TfR1 via receptor-mediated endocytosis into malignant cells [67]. Moreover, by simultaneously targeting NCOA4 and depleting the amount of free iron in neoplastic cells, it could be possible to further reduce cell growth and proliferation rates.

## 5. Conclusions

The results of this study, even if performed on a limited number of samples, preliminarily describe the expression of iron-related proteins in non-neoplastic testes and underscore the significance of TfR1 and NCOA4 expression in canine neoplastic testes. Future studies analyzing a bigger sample number and performing a more in-depth evaluation of quantitative protein expression will further corroborate the IHC results of the present study. Moreover, by transposing this study in 2D cell lines and 3D organoid models and performing knockdowns and/or overexpressions of the aforementioned proteins, it will be possible to dynamically understand the exact mechanisms underlying the observed changes in the metabolic pathways. The altered expression of iron metabolism-related proteins in almost all of the canine testicular neoplasia suggests their significance as potential therapeutic targets; therefore, the delivery of transferrin-chemotherapic conjugates via TfR1 should be considered in canine testicular cancers.

Moreover, by targeting iron metabolism and reducing uptake and mobilization from Ft storage, it could be possible to decrease proliferation rates and tumor growth.

## Figures and Tables

**Table 1:** Primary antibodies used for immunohistochemistry analysis.

Antibody	Manufacturer/Clone	Host Species	RIDD Number	Working Concentration	Dilution
TfR1	ThermoFisher, Carlsbad, CA, USA H68.4	Mouse	AB_2533029	0.05 mg/mL	1:100
FTH1	Antibodies, Limerick, PA, USA/Polyclonal	Rabbit	AB_3351669	0.05 mg/mL	1:100
NCOA4	Abcam, Cambridge, UK 439CT10.1.2	Mouse	AB_3076582	0.005 mg/mL	1:100
PCNA	ThermoFisher, Carlsbad, CA, USA PC10	Mouse	AB_10374347	0.2 mg/mL	1:400

**Table 2:** Primary antibodies used in Western blot analysis.

Antibody	Manufacturer/Clone	Host Species	Dilution
NCOA4	Abcam, Cambridge, UK 439CT10.1.2	Mouse	1:500
FTH1	Antibodies, Limerick, PA, USA/Polyclonal	Rabbit	1:500

**Table 3:** Immunoreactivity scoring of TfR1, NCOA4, FTH1 and PCNA in 5 canine SCT samples, 5 canine ITSEM samples, 13 canine DSEM samples, 2 canine LCT samples and 3 canine non-neoplastic testis samples.

Sample	Histology	TfR1	NCOA4	FHT1	PCNA
S1	SCT	-, 0	++, 2	+, 1	+, 1
S2	SCT	-, 0	+, 1	-, 0	+, 1
S3	SCT	-, 0	++, 2	-, 0	++, 2
S4	SCT	+, 1	++, 2	-, 0	+, 1
S5	SCT	-, 0	++, 2	-, 0	++, 2
S6	ITSEM	+, 1	+, 1	++, 2	++++, 4
S7	ITSEM	+, 1	+, 1	+++, 3	++++, 4
S8	ITSEM	+, 1	-, 0	+++, 3	+++, 3
S9	ITSEM	+, 1	+, 1	++, 2	+++, 3
S10	ITSEM	+, 1	+, 1	+++, 3	++, 2
S11	DSEM	+++, 3	++, 2	+, 1	++, 2
S12	DSEM	++, 2	++, 2	+, 1	+++, 3
S13	DSEM	+++, 3	+++, 3	++, 2	+, 1
S14	DSEM	+++, 3	+++, 3	++, 2	+++, 3
S15	DSEM	++, 2	++, 2	++, 2	+++, 3
S16	DSEM	++++, 4	++, 2	+, 1	+++, 3
S17	DSEM	++++, 4	++, 2	+, 1	++, 2
S18	DSEM	++++, 4	++, 2	++, 2	+++, 3
S19	DSEM	+++, 4	+++, 3	++, 2	++++, 4
S20	DSEM	+++, 3	++, 2	+, 1	++++, 4
S21	DSEM	+++, 3	++, 2	++, 2	++, 2
S22	LCT	++, 2	++, 2	+, 1	+++, 3
S23	LCT	+++, 3	++, 2	++, 2	+++, 3
N1	N.n. testis	+++, 3	++, 2	+, 1	++, 2
N2	N.n. testis	++, 2	+, 1	++, 2	+, 1
N3	N.n. testis	+++, 3	++, 2	+, 1	++, 2

SCT: Sertoli cell tumor; ITSEM: intratubular seminoma; DSEM: diffuse seminoma; LCT: Leydig cell tumor; N.n.: non-neoplastic; “-”: 0; “+” (<10%): 1; “++” (11–40%): 2; “+++” (41–80%): 3; “++++” (81–100%): 4.

## Data Availability

Further information on the data included in this study is available from the corresponding author upon reasonable request.

## Supplementary Materials

The following supporting information can be downloaded at: https://www.mdpi.com/article/10.3390/ani14172619/s1, Figure S1: Densitometric analysis of FTH1 (A) and NCOA4 (B) protein expression levels. The mean ± SEM of the densitometric values for the bands, each normalized on the relative densitometric values of β-Actin, are reported in the histogram. Protein levels of non neoplastic testis 1 sample were chosen as reference. Experimental replicates = 2. N.n. testis: non neoplastic testis; Table S1: Breed, age and histological findings of 26 canine testis.
