# Supplementary material for: Preliminary Evidence of the Possible Roles of the Ferritinophagy-Iron Uptake Axis in Canine Testicular Cancer
Source: Animals (Basel). 2024 Sep 9;14(17):2619. doi: 10.3390/ani14172619 (PMC11394645; doi:10.3390/ani14172619)
Supplement: Supplementary file 1 [file animals-14-02619-s001.zip › Figure S1.pdf]

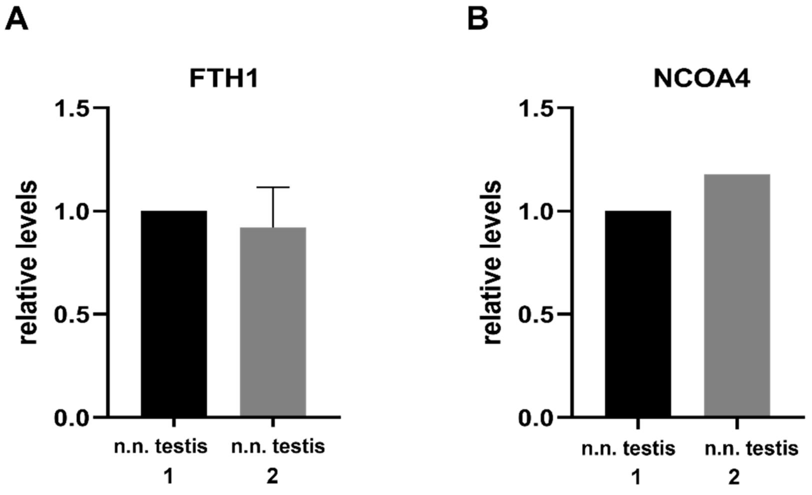

Figure S1. Densitometric analysis of FTH1 (A) and NCOA4 (B) protein expression levels. The mean ± SEM of the densitometric values for the bands, each normalized on the relative densitometric values of β-Actin, are reported in the histogram. Protein levels of non neoplastic testis 1 sample were chosen as reference. Experimental replicates=2. N.n. testis: non neoplastic testis.
