# Supplementary material for: Preliminary Evidence of the Possible Roles of the Ferritinophagy-Iron Uptake Axis in Canine Testicular Cancer
Source: Animals (Basel). 2024 Sep 9;14(17):2619. doi: 10.3390/ani14172619 (PMC11394645; doi:10.3390/ani14172619)
Supplement: Supplementary file 1 [file animals-14-02619-s001.zip › full blot.pdf]

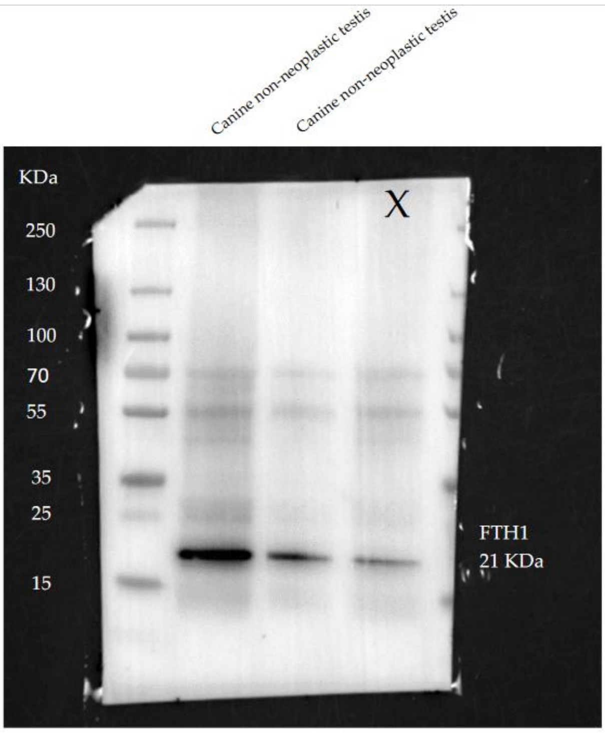

Full blot FTH1: Representative immunoblot analysis of FTH1 (21 KDa) performed on total protein lysates of two non-neoplastic canine testis samples.

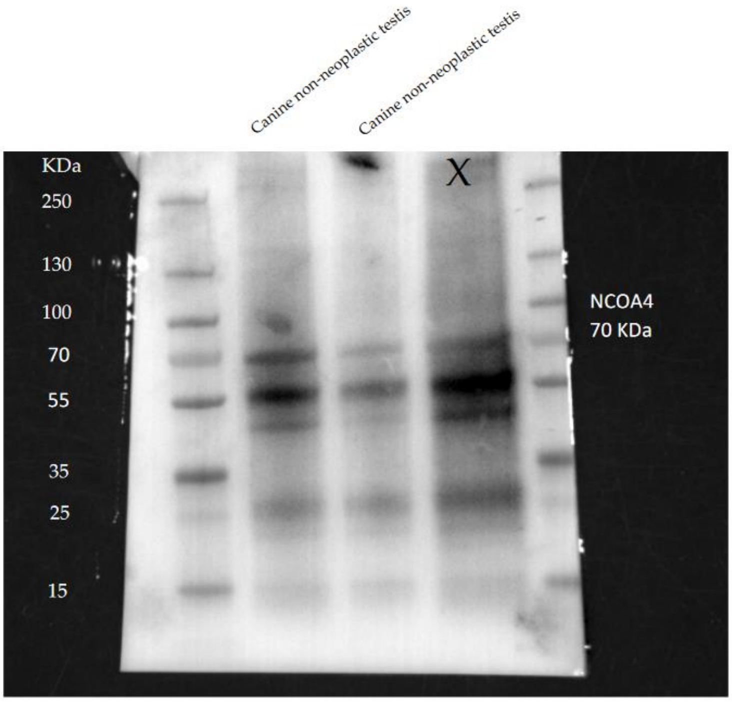

Full blot NCOA4: Representative immunoblot analysis of NCOA4 (70 KDa) performed on total protein lysates of two non-neoplastic canine testis samples.

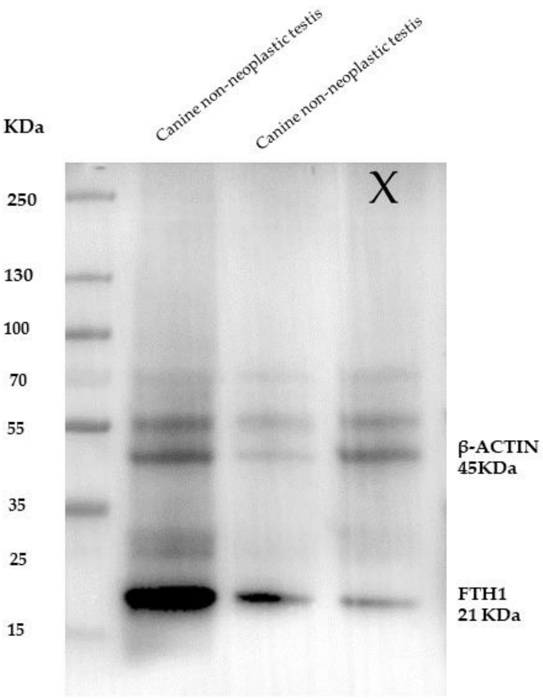

Rapresentative immunoblot analysis of β –Actin (45 KDa) performed on total protein lysates of two non-neoplastic canine testis samples in a stripped blot. 60μg of lysates were loaded for each sample, whose tag is reported on the right of the membrane.
