# Supplementary material for: Preliminary Evidence of the Possible Roles of the Ferritinophagy-Iron Uptake Axis in Canine Testicular Cancer
Source: Animals (Basel). 2024 Sep 9;14(17):2619. doi: 10.3390/ani14172619 (PMC11394645; doi:10.3390/ani14172619)
Supplement: Supplementary file 1 [file animals-14-02619-s001.zip › Table S1.pdf]

Table S1. Breed, age and histological findings of 26 canine testis.

Sample	Breed	Age (ys)	Histological findings
S1	Poodle	5	SCT
S2	English Setter	9	SCT
S3	English Setter	9	SCT
S4	Mixed breed	6	SCT
S5	German Shepherd	7	SCT
S6	Mixed breed	12	ITSEM
S7	Mixed breed	10	ITSEM
S8	Pitbull	12	ITSEM
S9	Mixed breed	11	ITSEM
S10	Mixed breed	11	ITSEM
S11	Beagle	11	DSEM
S12	German Shepherd	8	DSEM
S13	German Shepherd	10	DSEM
S14	Mixed breed	6	DSEM
S15	Mixed breed	16	DSEM
S16	West highland Terrier	7	DSEM
S17	Poodle	5	DSEM
S18	English Setter	9	DSEM
S19	English Setter	9	DSEM
S20	Mixed breed	6	DSEM
S21	German Shepherd	7	DSEM
S22	Mixed breed	9	LCT
S23	German Shepherd	10	LCT
N1	Poodle	8	N.n. testis
N2	German Shepherd	9	N.n. testis
N3	English Setter	7	N.n. testis

SCT: Sertoli cell tumor; ITSEM: Intratubular Seminoma; DSEM: Diffuse Seminoma; LCT: Leydig cell tumor; N.n. testis: Non-neoplastic testis.
